# Admission lactate at ICU entry and the risk of postoperative delirium after cardiac surgery: a retrospective cohort study using the eICU-CRD database

**DOI:** 10.1186/s12872-026-05915-5

**Published:** 2026-05-07

**Authors:** Qiankun Yang, ZhenGuo Luo, Jing Li, Gang Wu

**Affiliations:** 1 Department of Anesthesiology, Honghui Hospital, Xi’an Jiaotong University, Xi’an, Shaanxi China; 2 Department of Critical Care Medicine, Xi’an People’s Hospital (Xi’an Fourth Hospital), Affiliated People’s Hospital of Northwest University, Xi’an, Shaanxi China

**Keywords:** Lactate, Postoperative delirium, Cardiac surgery, Risk prediction, ICU outcomes

## Abstract

**Background:**

Postoperative delirium is a frequent complication after cardiac surgery. The predictive value of initial lactate at ICU admission for cardiac surgery–associated postoperative delirium (CS-POD) remains unclear.

**Methods:**

We conducted a retrospective cohort study using the eICU Collaborative Research Database, including adult patients admitted to the ICU after cardiac surgery. Multivariable logistic regression, restricted cubic spline analysis, ROC analysis, and mediation analysis were performed to assess the association between initial lactate and CS-POD and to explore whether disease severity scores (SOFA, APACHE IV) or clinical interventions statistically explained this relationship.

**Results:**

Among 358 patients, 104 (29.1%) developed CS-POD. Higher initial lactate at ICU admission was independently associated with increased risk of CS-POD after full adjustment (OR per 1 mmol/L increase: 1.37, 95% CI 1.11–1.69; *P* = 0.003). Restricted cubic spline analysis demonstrated a linear relationship between initial lactate and CS-POD risk (P for nonlinearity = 0.132). Adding initial lactate to the baseline prediction model improved discriminative performance, with the area under the ROC curve (AUC) increasing from 0.661 (95% CI, 0.598–0.723) to 0.717 (95% CI, 0.660–0.775) (DeLong test *P* = 0.012). The optimal lactate cutoff for predicting CS-POD was 2.385 mmol/L. Mediation analysis indicated that part of the association between initial lactate and CS-POD was statistically explained by SOFA (proportion statistically explained: 17%, *P* = 0.002) and APACHE IV (proportion statistically explained: 7.7%, *P* = 0.034), whereas clinical interventions (IABP, RRT, opioid use) did not show significant mediation. Higher initial lactate levels were also associated with increased risks of acute kidney injury (OR 1.27, 95% CI 1.00–1.61) and in-hospital mortality (OR 2.88, 95% CI 1.52–5.46).

**Conclusions:**

Higher initial lactate at ICU admission is associated with an increased risk of CS-POD and adverse postoperative outcomes. However, its predictive value is modest and should be interpreted cautiously as part of a broader clinical assessment rather than as an independent decision-making tool.

**Supplementary Information:**

The online version contains supplementary material available at 10.1186/s12872-026-05915-5.

## Introduction

Delirium is an acute neurocognitive disorder characterized by fluctuating attention, awareness, and cognition. Postoperative delirium (POD) occurs frequently in older adults following cardiac surgery, with reported incidences reaching up to 50% [1]. It is associated with increased morbidity and mortality, longer intensive care unit (ICU) and hospital stays, and long-term cognitive decline [2]. Patients who develop POD are also at higher risk of persistent cognitive impairment for up to a year after surgery [3].

The pathogenesis of POD is complex and multifactorial, encompassing preoperative cognitive deficits, intraoperative hemodynamic instability, the use of cardiopulmonary bypass (CPB), systemic inflammation, ischemia–reperfusion injury, and postoperative pain management strategies [3, 4]. Neuroinflammation has been increasingly recognized as a central mechanism, with systemic inflammatory mediators either crossing the blood–brain barrier or being produced locally in response to surgical stress [5]. In cardiac surgery, CPB and ischemia–reperfusion events may exacerbate neuroinflammation, further increasing the risk of POD [6]. Additionally, both inadequate pain control and excessive opioid administration have been implicated in delirium development [7, 8].

Despite growing awareness, the mechanisms underlying POD remain incompletely understood, and effective prophylactic strategies are limited. Pharmacologic interventions, including antipsychotics and sedatives, have produced inconsistent results, with recent meta-analyses suggesting that antipsychotics are largely ineffective in preventing or treating POD [9–11]. Consequently, there is increasing interest in identifying reliable early predictors of POD, particularly metabolic and physiological markers [12, 13].

Lactate, a key indicator of anaerobic metabolism, has emerged as a potential early prognostic marker for adverse postoperative outcomes [14]. Elevated lactate levels measured at ICU admission reflect tissue hypoperfusion, metabolic stress, and systemic inflammation, all of which may contribute to neurocognitive dysfunction [15, 16]. In the context of cardiac surgery, where CPB and ischemia–reperfusion events can induce acute metabolic disturbances, admission lactate may serve as a valuable predictor of POD risk [17]. However, the association between lactate levels and POD remains inadequately explored.

In this multicenter study, we analyzed data from multiple ICUs across different hospitals to evaluate whether initial lactate at ICU admission provides additional predictive value for cardiac surgery–associated postoperative delirium (CS-POD) beyond demographic and preoperative factors. We compared a baseline risk model with a lactate-enhanced model and also assessed secondary outcomes, including ICU length of stay, hospital length of stay, and in-hospital mortality, to comprehensively evaluate the clinical impact of admission lactate.

## Method and materials

### Sources of data and ethics compliance

This is a retrospective observational study. The Philips eICU program is a transformational critical care telehealth program that provides 24-h support for caregivers at the bedside. The eICU-CRD v2.0 encompasses more than 200,859 ICU admissions of 139,367 unique patients from 208 hospitals across the United States in 2014 and 2015 [18]. This is a publicly available critical care database that includes detailed clinical information from patients admitted to intensive care units (ICUs) across multiple healthcare facilities in the United States. The eICU-CRD includes a wide range of patient data, such as demographic information, vital signs, laboratory results, medications, and outcomes, collected during ICU admissions. All data in the eICU-CRD are anonymized to ensure patient privacy, and the database was approved for use in research by the institutional review board (IRB) at the Massachusetts Institute of Technology (MIT). As this study utilizes de-identified publicly available data, it is exempt from the requirement for individual patient consent. The research was conducted in accordance with relevant ethical guidelines and regulations governing the use of secondary data for research purposes. The database comprises de-identified critical care data from multiple hospitals in the United States. Data were accessed on 20/12/2021 for research purposes.

### Patients

**Fig. 1:**
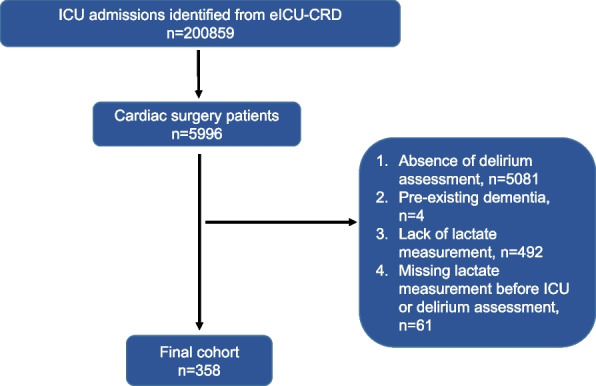
Flowchart of patient inclusion and exclusion

Data for this study were obtained from the eICU Collaborative Research Database (eICU-CRD), which included 200,859 ICU admissions. From this population, 5,996 patients underwent cardiac surgery. Patients were excluded for the following reasons: absence of documented delirium assessments during the ICU stay (*n* = 5,081), pre-existing dementia (*n* = 4), missing lactate measurements (*n* = 492), or lactate measurements not obtained prior to ICU admission or delirium assessment (*n* = 61). After applying these criteria, a total of 358 patients were included in the final cohort (Fig. 1). All patients were followed throughout their ICU stay to assess the occurrence of postoperative delirium and other clinical outcomes. We additionally compared baseline characteristics between included and excluded patients using available variables in the database to assess potential selection bias.

### Data extraction

Data for this study were extracted from the eICU-CRD database, focusing on patients who underwent cardiac surgery and had postoperative lactate measurements. Procedures for cardiac surgery and diagnoses of postoperative delirium were extracted from the database using ICD-9 and ICD-10 codes, with the specific codes listed in Supplementary Table S1. Other demographic, clinical, and laboratory variables were extracted from the database using the same coding and data query procedures. The variables extracted included variables related to patient demographics, comorbidities, laboratory findings, severity of illness, and treatment/support within the first 48 h. Key variables extracted include: 1) Demographics: age, BMI, gender, ethnicity; 2) Comorbidities: hypertension, myocardial infarction, congestive heart failure, peripheral vascular disease, cerebrovascular disease, chronic pulmonary disease, mild liver disease, diabetes, renal disease, malignant cancer, stroke, and Charlson Comorbidity Index; 3) Laboratory data: maximum creatinine, maximum chloride, minimum hemoglobin, minimum platelet count, and maximum blood urea nitrogen (BUN); 4) Severity of Illness: SOFA score and APACHE IV score; 5) Support within the First 48 Hours: hemodialysis, intra-aortic balloon pump (IABP), and opioid administration; 6) Outcomes and Prognosis: delirium score (primary outcome), in-hospital mortality, acute kidney injury (AKI, KDIGO criteria), renal replacement therapy (RRT included any modality of dialysis (intermittent hemodialysis, continuous renal replacement therapy [CRRT], or sustained low-efficiency dialysis), ICU length of stay, and total length of hospital stay (secondary outcomes). Postoperative lactate measurements obtained at ICU admission were extracted for analysis. The initial lactate value measured upon ICU admission was used, as it reflects the patient’s early metabolic status immediately after surgery and may provide prognostic information regarding postoperative delirium and other adverse outcomes. The median time at which the maximum lactate value was recorded was 6.32 h (IQR, 1.87–21.25 h) postoperatively (ICU admmision). Serial lactate trajectories were not analyzed due to heterogeneity in measurement frequency and timing across centers.

Missing data for variables with < 20% missingness were imputed using the “predictive mean matching (PMM)” method implemented in the mice R package, with 5 imputed datasets generated. Outliers were identified using statistical methods and extreme values were truncated based on the distribution characteristics of each variable, assuming approximate normality for most data. The proportion of missing values for each variable is presented in Supplementary Figure S1, showing that only a few variables had missing data, and most key variables were largely complete. To assess the robustness of our findings to the handling of missing data, we performed a complete case analysis, including only patients with no missing values for any covariates used in the fully adjusted model. Logistic regression models identical to the primary analysis were applied, and results were compared with those obtained using multiple imputation.

### Outcomes and assessment

The primary outcome of this study was the incidence of postoperative delirium, which was assessed using the Confusion Assessment Method for the ICU (CAM-ICU) and the Intensive Care Delirium Screening Checklist (ICDSC). Assessments were performed by trained ICU nursing staff as part of routine clinical care, typically once per nursing shift (approximately every 8–12 h). Both CAM-ICU and ICDSC have been validated in critically ill populations for reliable detection of delirium. We acknowledge that intermittent assessments may underestimate transient or short-duration delirium episodes. Both tools have demonstrated high sensitivity and specificity in delirium diagnosis, with bivariate meta-analysis showing consolidated sensitivities of 0.84 for CAM-ICU and 0.83 for ICDSC, and consolidated specificities of 0.95 and 0.87, respectively. These delirium assessment tools are comprehensively documented in the eICU-CRD database and were used to reliably identify cases of delirium in the study cohort.

Prior to delirium assessment, the Richmond Agitation-Sedation Scale (RASS) was evaluated to determine the patient’s level of arousal; patients with RASS ≤ –3 were considered unassessable at that time. When CAM-ICU and ICDSC results were discordant, a positive result on either scale was used to classify the patient as delirious, consistent with prior studies. Patients who could not be assessed due to deep sedation or coma were recorded, and the proportion of unassessable assessments is reported in the Results section. Both CAM-ICU and ICDSC have been validated in critically ill populations, with high sensitivity and specificity.

### Statistical analysis

The study followed the Strengthening the Reporting of Observational Studies in Epidemiology (STROBE) guidelines [19]. First, a descriptive statistical overview and differences between the delirium and control groups were examined. The Shapiro–Wilk test was used to assess the normality of continuous variables, which indicated that most variables did not follow a normal distribution. Continuous variables were therefore presented as medians with interquartile ranges (IQRs), while categorical variables were expressed as frequencies and percentages. To detect differences between the delirium and control groups, the Kruskal–Wallis test was applied for continuous variables and the chi-squared test for categorical variables.

Lactate was primarily analyzed as a continuous variable, with additional analyses performed using a dichotomized cutoff derived from ROC analysis. Logistic regression was utilized to evaluate the association between lactate levels and delirium, providing odds ratios (OR) and 95% confidence intervals (CI). Length of stay outcomes (ICU and hospital) were analyzed using linear regression models after log-transformation due to their skewed distribution. Results are presented as regression coefficients and interpreted as relative percentage changes. Five levels of multivariate adjustment were applied to the models. Model I was adjusted for demographic variables, including lactate group, age, BMI, gender, and ethnicity. Model II included the same variables as Model I, with additional adjustment for comorbidities such as hypertension, myocardial infarction, congestive heart failure, peripheral vascular disease, cerebrovascular disease, chronic pulmonary disease, mild liver disease, diabetes, renal disease, malignant cancer, stroke, and the Charlson comorbidity index. Model III further adjusted for clinical parameters, including creatinine levels, chloride, hemoglobin, platelet count, blood urea nitrogen (BUN), while retaining the adjustments from Model II. Model IV additionally adjusted for treatment and intervention measures such as hemodialysis, intra-aortic balloon pump (IABP), and opioid use. Backward stepwise logistic regression was applied to select the most optimal predictive model, based on the lowest Akaike Information Criterion (AIC). The variance inflation factor (VIF) was used to identify and exclude continuous variables with a VIF greater than 5 (Supplementary Table S2). To evaluate the robustness of our findings, additional sensitivity analyses were performed excluding patients with high postoperative opioid exposure, using the same multivariable logistic regression models as the primary analysis. Model calibration was evaluated using the Hosmer–Lemeshow test and calibration plots, to assess the agreement between predicted and observed probabilities.

To explore the nonlinear relationship between lactate levels and delirium, we applied restrictive cubic splines. This method allows for the modeling of potential non-linear associations by fitting a series of knots at predefined percentiles of lactate levels. The analysis was conducted to visualize and assess how variations in lactate levels influence the odds of delirium, providing a more nuanced understanding of the association beyond linear effects.

We performed mediation analysis to explore whether illness severity scores (SOFA and APACHE IV) or clinical interventions statistically explained part of the association between admission lactate and postoperative delirium. Given the observational design, this analysis should be interpreted as a statistical decomposition of associations rather than causal mediation. The estimation of average causal mediation effects (ACME) relies on assumptions such as no unmeasured confounding between exposure, mediator, and outcome, which may not be fully satisfied in this setting.

To assess the incremental predictive value of lactate for delirium, the area under the receiver operating characteristic curve (AUC) was calculated for each model. The AUC quantifies the predictive accuracy of the model, with higher values indicating better discrimination between delirium and non-delirium cases. The DeLong method was applied to compare the AUCs before and after the inclusion of lactate, enabling us to evaluate the added predictive value of lactate in the prediction of delirium. All statistical analyses were conducted using R version 4.3.1 (R Foundation for Statistical Computing, Vienna, Austria). Key packages utilized included *dplyr* for data manipulation, *ggplot2* for data visualization, *rms* for regression modeling and calibration analysis, *pROC* for receiver operating characteristic (ROC) curve and AUC calculations.

## Results

### Baseline characteristics of participants

Among the 358 patients included, 104 (29.1%) developed cardiac surgery–associated postoperative delirium (CS-POD). Median age was 65 years [IQR, 56–73] in the non-delirium group and 66 years [IQR, 58–76] in the delirium group; median BMI was similar between groups (27.98 vs 28.14 kg/m2). The distribution of sex and ethnicity did not differ significantly. Patients who developed delirium were more likely to have undergone combined surgery—defined as receiving both coronary artery bypass grafting (CABG) and valve surgery in the same operative procedure (12.5% vs 4.7%)—and had higher rates of congestive heart failure (33.7% vs 16.9%), as well as higher APACHE IV (68 vs 58), CCI (4 vs 4), and SOFA (6 vs 5) scores. Laboratory values on ICU admission showed higher creatinine (1.27 vs 1.02 mg/dL), BUN (20.0 vs 17.0 mg/dL), and lower hemoglobin (7.75 vs 9.10 g/dL) and platelets (102.5 vs 132.5 × 10⁹/L) among patients with delirium. Vasopressor use was more frequent in the delirium group (49.0% vs 31.5%), whereas opioid use did not differ. Delirium was associated with worse outcomes, including higher in-hospital mortality (13.5% vs 2.4%), higher incidence of AKI (33.7% vs 13.8%), and longer ICU (5.56 vs 1.95 days) and hospital length of stay (15.09 vs 8.36 days) (Table 1). A comparison between included and excluded patients is shown in Supplementary Table S3. Overall, baseline characteristics were broadly comparable between the two groups, although included patients tended to have slightly higher illness severity scores and lactate levels, suggesting a potential but limited selection bias.

**Table 1:** Baseline characteristics of patients stratified by postoperative delirium status

Variables	Non-delirium group (*n* = 254)	Delirium group (*n* = 104)	*P*
Age (years)	65.00 [56.00, 73.00]	66.00 [58.00, 76.00]	0.1505
BMI (kg/m^2^)	27.98 [24.58, 32.19]	28.14 [24.09, 35.15]	0.4271
Male	179 (70.47%)	66 (63.46%)	0.2418
Ethnicity			0.6276
White	161 (63.39%)	62 (59.62%)	
Black	54 (21.26%)	27 (25.96%)	
Other or Unknown	39 (15.35%)	15 (14.42%)	
Type of Surgery			0.025
CABG	141 (55.51%)	49 (47.12%)	
Valve surgery	101 (39.76%)	42 (40.38%)	
Combined Surgery#	12 (4.72%)	13 (12.50%)	
Comorbidities			
Hypertension	56 (22.05%)	31 (29.81%)	0.156
Myocardial infarction	59 (23.23%)	26 (25.00%)	0.8252
Heart failure	43 (16.93%)	35 (33.65%)	0.0008
Peripheral vascular disease	14 (5.51%)	12 (11.54%)	0.0766
Cerebrovascular disease	22 (8.66%)	10 (9.62%)	0.9337
Chronic pulmonary disease	32 (12.60%)	19 (18.27%)	0.2198
Severe liver disease	2 (0.79%)	3 (2.88%)	0.2987
Diabetes	80 (31.50%)	29 (27.88%)	0.5839
Renal disease	40 (15.75%)	24 (23.08%)	0.1359
Malignant cancer	16 (6.30%)	8 (7.69%)	0.8059
Stroke	2 (0.79%)	3 (2.88%)	0.2987
Laboratory data, median(IQR)^a^			
Creatinine (mg/dL)	1.02 [0.83, 1.33]	1.27 [0.97, 1.64]	0.0001
Hemoglobin (g/dL)	9.10 [7.62, 10.57]	7.75 [6.90, 9.40]	< 0.001
Platelets (109/L)	132.50 [100.00, 171.00]	102.50 [72.75, 153.75]	0.0006
BUN (mg/dL)	17.00 [13.00, 23.75]	20.00 [16.00, 31.00]	0.0004
Severity of illness, median(IQR)^b^			
APACHE IV	58.00 [40.00, 78.75]	68.00 [53.00, 87.00]	0.0005
CCI	4.00 [3.00, 5.00]	4.00 [3.00, 6.00]	0.0012
SOFA	5.00 [3.00, 6.75]	6.00 [4.00, 8.00]	< 0.001
Support within the first 48 h			
Opioid Use	130 (51.18%)	49 (47.12%)	0.5605
Vasopressor Use	80 (31.50%)	51 (49.04%)	0.0026
IABP use	13 (5.12%)	12 (11.54%)	0.0529
Outcomes and Prognosis			
In-Hospital Mortality	6 (2.36%)	14 (13.46%)	0.0001
RRT	10 (3.94%)	5 (4.81%)	0.934
AKI	35 (13.78%)	35 (33.65%)	< 0.001
Total Hospital Length of Stay (days)	8.36 [6.34, 11.61]	15.09 [9.26, 24.53]	< 0.001
ICU Length of Stay (days)	1.95 [1.23, 3.75]	5.56 [2.88, 11.11]	< 0.001

*BMI* Body Mass Index, *CABG* Coronary Artery Bypass Grafting, *BUN* Blood Urea Nitrogen, *APACHE IV* Acute Physiology and Chronic Health Evaluation IV, *CCI* Charlson Comorbidity Index, *IABP* Intra-Aortic Balloon Pump, *RRT* Renal Replacement Therapy, *AKI* Acute kidney injury, *ICU* Intensive care unit

^a^Maximum or minimum value indicating disease severity within 24 h after ICU admission

^b^The first values during the first day after ICU admission

^#^Combined surgery refers to patients who underwent both coronary artery bypass grafting (CABG) and valve surgery during the same operative procedure

### Association between initial icu lactate and postoperative delirium

**Fig. 2:**
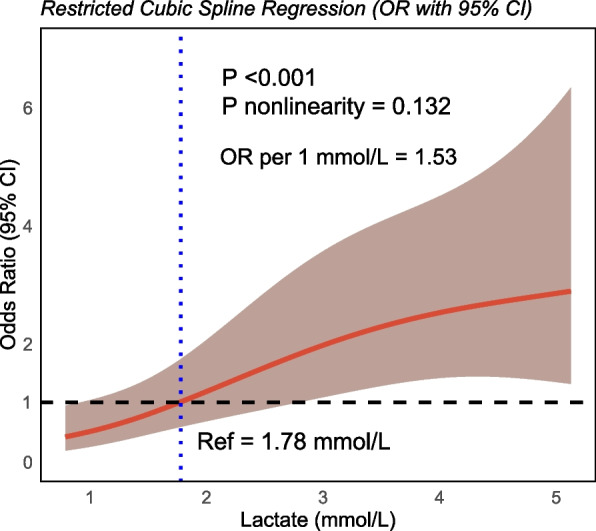
Restricted cubic spline analysis of the relationship between lactate levels and postoperative delirium

Higher initial lactate at ICU admission was significantly associated with increased risk of CS-POD. In multivariable logistic regression, the odds ratios (OR) per 1 mmol/L increase in lactate ranged from 1.37 (95% CI, 1.11–1.69; *P* = 0.003) in the fully adjusted model to 1.56 (95% CI, 1.30–1.88; *P* < 0.001) in the unadjusted model (Table 2). Restricted cubic spline analysis indicated a linear relationship between initial lactate and delirium risk (P for nonlinearity = 0.132), with no evidence of threshold effects (Fig. 2). In the complete case analysis, the association between initial lactate and postoperative delirium remained consistent with the primary analysis. In the fully adjusted model (Model IV), each 1 mmol/L increase in lactate was associated with an OR of 1.36 (95% CI, 1.11–1.70; *P* = 0.004), similar to the multiple imputation results. Detailed results are shown in Supplementary Table S4.

**Table 2:** Association between lactate levels and postoperative delirium risk

Model	OR	Lower 95%CI	Upper 95%CI	*P* Value
Model I	1.559	1.301	1.877	< 0.001
Model II	1.516	1.257	1.837	< 0.001
Model III	1.435	1.174	1.76	< 0.001
Model IV	1.37	1.113	1.692	0.003

Adjusted covariates

Model I = Demographic information including age, BMI, gender, and ethnicity

Model II = Model I + additional health status variables such as hypertension, myocardial infarction, congestive heart failure, peripheral vascular disease, cerebrovascular disease, chronic pulmonary disease, mild liver disease, diabetes, renal disease, malignant cancer, stroke, and Charlson comorbidity index

Model III = Model II + additional clinical indicators including maximum creatinine, maximum chloride, minimum hemoglobin, minimum platelet count, and maximum blood urea nitrogen (BUN)

Model IV = Model III + treatments and interventions such as hemodialysis, intra-aortic balloon pump (IABP), and opioid use

### Predictive performance of initial lactate for CS-POD

**Fig. 3:**
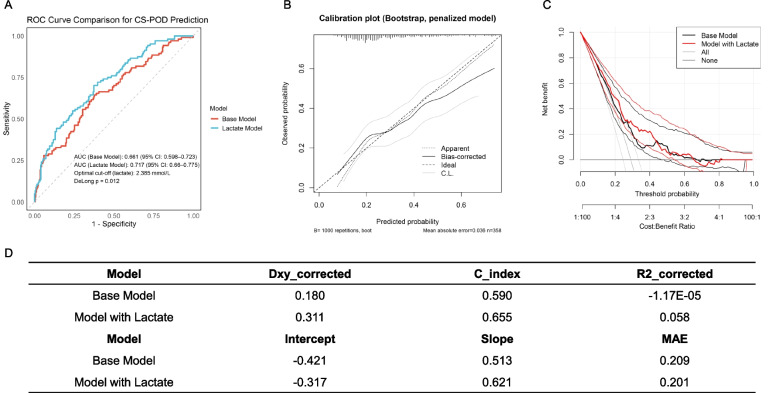
Predictive value of lactate for postoperative delirium after cardiac surgery. **A** Receiver operating characteristic (ROC) curves comparing the incremental predictive performance of lactate added to the baseline model. **B** Calibration curves of the prediction models. **C** Decision curve analysis demonstrating the clinical net benefit of models with and without lactate. **D** Bootstrap validation results of model performance. Comparison of ROC curves for CS-POD prediction: base model vs. lactate model, one adjusted for demographic variables and preoperative comorbidities, and the other adding lactate, with model performance assessed using the DeLong test

The addition of initial lactate at ICU admission improved the prediction of postoperative delirium compared with a baseline model including demographic and preoperative factors. In receiver operating characteristic (ROC) analysis, the baseline model had an AUC of 0.661 (95% CI, 0.598–0.723), which increased to 0.717 (95% CI, 0.660–0.775) after adding lactate (DeLong test *P* = 0.012) (Fig. 3A). This AUC gain is statistically significant but modest. Calibration curves demonstrated good agreement between predicted and observed CS-POD probabilities in the lactate-enhanced model (Fig. 3B). Decision curve analysis indicated that incorporating lactate provided a higher net clinical benefit across a range of threshold probabilities compared with the baseline model (Fig. 3C). Bootstrap validation demonstrated improved model performance with lactate: the corrected Dxy increased from 0.180 to 0.311, the C-index from 0.590 to 0.655, R2 from near zero (−1.17 × 10⁻5) to 0.058, and mean absolute error (MAE) decreased from 0.209 to 0.201, indicating enhanced discrimination and calibration (Fig. 3D). The optimal cutoff for admission lactate to predict CS-POD was 2.385 mmol/L. Patients with lactate above this threshold had significantly higher odds of developing delirium, with an OR of 2.91 (95% CI, 1.81–4.70; *P* < 0.001) in univariable analysis and 2.82 (95% CI, 1.70–4.69; *P* < 0.001) after full adjustment for demographic, comorbidity, laboratory, severity, and treatment variables (Table 3).

**Table 3:** Logistic regression analysis of postoperative delirium according to lactate dichotomized by ROC-derived cut-off

Model	OR	Lower 95%CI	Upper 95%CI	*P*
Univariable Model	2.908	1.808	4.6978	< 0.001
Full adjusted Model	2.816	1.7018	4.6858	< 0.001

Full adjusted Model were adjusted for the covariates listed, with Model IV in Table 2 including the full set of adjustments

### Association between initial lactate and secondary outcomes

Elevated initial lactate was associated with higher risks of adverse postoperative outcomes. In fully adjusted models, each 1 mmol/L increase in lactate was associated with increased odds of acute kidney injury (OR, 1.27; 95% CI, 1.00–1.61; *P* = 0.047) and in-hospital mortality (OR, 2.88; 95% CI, 1.52–5.46; *P* = 0.001), each 1 mmol/L increase in lactate was associated with a 13% increase in ICU length of stay and an 8% increase in hospital length of stay. Renal replacement therapy (RRT) was not significantly associated with lactate levels (OR, 0.79; 95% CI, 0.31–2.01; *P* = 0.623) (Table 4).

**Table 4:** Multivariable analysis of the association between lactate levels and secondary outcome prognostic indicators

Secondary outcomes	Effect estimate	Lower 95%CI	Upper 95%CI	*P*
AKI	OR = 1.270	1.003	1.609	0.047
RRT	OR = 0.792	0.311	2.012	0.623
In-hospital mortality	OR = 2.878	1.518	5.456	0.001
ICU Length of Stay†	β = 0.125	0.078	0.173	< 0.001
Length of hospital stay†	β = 0.077	0.038	0.116	< 0.001

AKI, RRT, and in-hospital mortality were analyzed using multivariable logistic regression and are presented as odds ratios (ORs)

^†^ ICU and hospital length of stay were analyzed using linear regression models after log-transformation due to their skewed distribution. Regression coefficients (β) represent the change in log-transformed length of stay per 1 mmol/L increase in lactate. These correspond approximately to a 13.3% increase in ICU length of stay and a 8.0% increase in hospital length of stay per unit increase in lactate

All models were adjusted for the covariates listed in the fully adjusted model (Model IV in Table 2)

### Mediation analysis of clinical indicators

Mediation analysis showed that part of the association between initial lactate and CS-POD was explained by illness severity scores, while clinical interventions did not significantly mediate the relationship. Specifically, SOFA score statistically explained 17% of the effect (average causal mediation effect [ACME] 0.01; 95% CI, 0.003–0.018; *P* = 0.002), and APACHE IV score statistically explained 7.7% (ACME 0.004; 95% CI, 0.001–0.01; *P* = 0.034). Other mediators, including IABP, RRT, and opioid use, did not show significant mediation (all *P* > 0.05) (Table 5).

**Table 5:** Mediation analysis results of different clinical indicators on the outcome, including total effect, average causal mediation effect (ACME), average direct effect (ADE), and proportion statistically explained

Mediator	Effect	Estimate	95% CI Lower	95% CI Upper	*p*-value
SOFA	Total Effect	0.058	0.041	0.071	< 0.001
	ACME (average)	0.01	0.003	0.018	0.002
	ADE (average)	0.048	0.029	0.064	< 0.001
	Prop. Statistically explained (average)	0.17	0.061	0.339	0.002
APACHE-IV	Total Effect	0.058	0.041	0.07	< 0.001
	ACME (average)	0.004	0.001	0.01	0.034
	ADE (average)	0.054	0.036	0.067	< 0.001
	Prop. Statistically explained (average)	0.077	0.008	0.191	0.034
IABP	Total Effect	0.056	0.041	0.07	< 0.001
	ACME (average)	0.001	−0.002	0.007	0.508
	ADE (average)	0.055	0.039	0.069	< 0.001
	Prop. Statistically explained (average)	0.013	−0.043	0.128	0.508
RRT	Total Effect	0.057	0.04	0.069	< 0.001
	ACME (average)	0.0002	−0.002	0.004	0.95
	ADE (average)	0.056	0.04	0.069	< 0.001
	Prop. Statistically explained (average)	0.0004	−0.044	0.065	0.95
Opioid use	Total Effect	0.056	0.041	0.069	< 0.001
	ACME (average)	0.0002	−0.003	0.004	0.906
	ADE (average)	0.056	0.041	0.069	< 0.001
	Prop. Statistically explained (average)	0.0009	−0.063	0.079	0.906

*Abbreviations* *SOFA* Sequential Organ Failure Assessment, *APACHE IV* Acute Physiology and Chronic Health Evaluation IV, *IABP* Intra-Aortic Balloon Pump, *RRT* Renal Replacement Therapy, *ACME* Average Causal Mediation Effect, *ADE* Average Direct Effect, *Prop* Statistically explained, Proportion Statistically explained

## Discussion

In this multicenter database study of cardiac surgery patients admitted to the ICU, we found that higher initial lactate levels at ICU admission were independently associated with an increased risk of cardiac surgery–associated postoperative delirium (CS‑POD). This association was evident in continuous models, as well as when lactate was dichotomized at an optimal threshold of 2.385 mmol/L, with higher lactate corresponding to significantly elevated odds of delirium. Moreover, adding admission lactate to a baseline prediction model modestly improved overall discriminative performance, suggesting that early metabolic perturbations may contribute incremental prognostic information beyond traditional demographic and clinical variables. In addition to delirium risk, elevated admission lactate was also associated with adverse clinical outcomes including acute kidney injury, in‑hospital mortality, and prolonged ICU and hospital stays, underscoring the potential utility of lactate as an integrative marker of early postoperative physiological stress.

Our findings resonate with prior evidence linking lactate dynamics to adverse perioperative outcomes. Although few studies have directly examined lactate in relation to postoperative delirium specifically after cardiac surgery, emerging data from a recent cohort study showed that peak lactate on the first postoperative day (maximum within 24 h) was significantly associated with POD in cardiac surgical patients, with a cutoff around 2.85 mmol/L identifying higher risk groups in multivariate analyses [20]. Additionally, previous work in noncardiac surgical populations and trauma patients has suggested that serum lactate at ICU admission or early postoperative periods may relate to delirium risk, though effect sizes and significance vary by context [21]. Collectively, these findings suggest that lactate, as a surrogate of tissue hypoperfusion and metabolic stress, may be a generalizable marker of vulnerability to delirium and other organ dysfunctions across surgical and critical care settings.

The pathophysiology of postoperative delirium is complex and multifactorial, involving systemic inflammation, neuroinflammation, endothelial dysfunction, and cerebral metabolic dysregulation. Surgical stress and cardiopulmonary bypass (CPB) can elicit robust inflammatory responses with elevated circulating cytokines and chemokines, which may disrupt the blood–brain barrier (BBB) and facilitate neuroinflammatory cascades [22]. A prospective cohort study investigating cerebrospinal fluid (CSF) biomarkers found that postoperative delirium was associated with blood–brain barrier breakdown and increased CSF lactate, supporting a link between metabolic disruption and neuroinflammation in delirium pathogenesis [23]. Moreover, systemic inflammatory mediators such as interleukin‑6 (IL‑6) and neutrophil‑lymphocyte ratios have been associated with delirium onset and severity in cardiac surgical settings, pointing toward a role for inflammatory pathways interacting with metabolic stress [24]. Lactate may participate in these processes not only as a byproduct of anaerobic metabolism but also as a signaling molecule influencing immune cell activation and regional cerebral perfusion, thereby interfacing with neuroinflammatory mechanisms.

In clinical practice, postoperative delirium represents a common and significant complication following cardiac surgery, with incidence rates reported widely but frequently exceeding 25% in ICU cohorts [25]. Delirium has been linked to longer lengths of stay, higher health care utilization, increased mortality, and long‑term cognitive deficits, emphasizing the importance of early risk identification and tailored interventions. Early metabolic markers such as admission lactate could be integrated into risk stratification tools to identify high‑risk patients for enhanced monitoring or preemptive strategies, including nonpharmacologic delirium prevention bundles that emphasize sleep hygiene, early mobilization, and cognitive stimulation. Furthermore, adjunctive pharmacologic approaches such as perioperative dexmedetomidine have shown some promise in reducing delirium incidence in cardiac surgery populations, although heterogeneity in study findings underscores the need for cautious interpretation [26].

Future research should explore the temporal dynamics of lactate and other metabolic markers in relation to delirium onset using time‑to‑event analyses with repeated measures, as well as incorporate broader sets of perioperative variables including inflammatory mediators, cerebral perfusion metrics, and intraoperative factors like CPB duration and blood pressure variability. Integrating metabolic, inflammatory, and neurophysiological data may yield more refined predictive models and provide mechanistic insights into delirium pathogenesis. Prospective validation in diverse cardiac surgical cohorts will be essential to establish the robustness and clinical utility of lactate‑based risk prediction.

In our study, the optimal cutoff of initial lactate for predicting postoperative delirium was 2.385 mmol/L. This value is slightly above the commonly recognized clinical threshold for hyperlactatemia of 2.0 mmol/L, which is widely used to identify patients at risk of tissue hypoperfusion and adverse outcomes. The proximity of our derived threshold to this established cutoff underscores its potential clinical relevance: patients with lactate levels above 2.0–2.4 mmol/L at ICU admission may warrant closer monitoring for delirium and other postoperative complications. While the exact value may vary between cohorts, our findings suggest that even modest elevations in lactate beyond standard clinical thresholds carry prognostic significance for neurocognitive outcomes after cardiac surgery.

Adjustment for severity scores (SOFA, APACHE IV) and postoperative interventions may lie on the causal pathway between admission lactate and postoperative delirium. Including these variables in multivariable models could attenuate the observed association, potentially underestimating the true effect of lactate. Although our mediation analysis provides additional insight into indirect pathways, we acknowledge that residual bias from pathway adjustment cannot be fully excluded.

### Limitations

This study has several limitations. First, its retrospective design limits causal inference, and although we used ICU admission lactate and excluded measurements after delirium assessment, temporal ambiguity and potential reverse causation cannot be fully excluded. Given the relatively small number of outcome events compared with the number of covariates included in the models, there is a potential risk of overfitting. Although we applied model selection and validation techniques, the observed performance metrics (C-index and calibration slope) indicate only modest model robustness. Therefore, the predictive performance of our model should be interpreted with caution, and external validation in larger cohorts is warranted. The mediation analysis in this study should be interpreted with caution. Although we applied standard methods to estimate ACME, these approaches rely on strong assumptions, including the absence of unmeasured confounding between exposure, mediator, and outcome. Given the retrospective observational design, these assumptions are unlikely to be fully met. Therefore, our findings should be understood as a statistical decomposition of the observed association rather than evidence of causal mediation pathways. Second, only the initial lactate measurement at ICU admission was considered, which may not capture dynamic lactate changes or clearance. Third, important intraoperative confounders such as cardiopulmonary bypass duration, cross-clamp time, perfusion pressures, transfusions, temperature, and anesthetic/sedative exposures were not available. Fourthly, a substantial proportion of patients (84.7%) were excluded, primarily due to the absence of documented delirium assessments. This may introduce significant selection bias, as patients who underwent delirium evaluation may differ systematically from those who did not. For example, excluded patients may have been either more severely ill (e.g., deeply sedated and therefore not assessable) or less closely monitored, which could influence both lactate levels and delirium risk. Although we performed a comparative analysis between included and excluded patients using available variables, residual differences cannot be excluded. Therefore, the generalizability of our findings may be limited. Our final analytic cohort included only 358 patients, representing a highly selected subset of the original cardiac surgery population due to missing delirium assessments. As shown in Table S3, included and excluded patients were generally comparable with respect to demographics, comorbidities, and admission lactate levels; however, the selective nature of the cohort and the relatively small sample size may limit the generalizability of our findings and introduce potential bias toward centers with structured delirium screening. Readers should interpret our results with these considerations in mind. Furthermore, delirium assessment was conducted across multiple centers with potentially heterogeneous practices. Despite the use of validated tools (CAM-ICU and ICDSC), variations in assessment frequency, staff training, sedation practices, and local protocols may have resulted in misclassification or under-detection of delirium. This heterogeneity in outcome ascertainment represents an important limitation. Finally, it is important to note that our study is based on data from the eICU-CRD collected between 2014 and 2015. Since then, advances in cardiac surgical techniques, perioperative care, ICU monitoring, and delirium prevention strategies may limit the direct generalizability of our findings to contemporary clinical practice. Therefore, while our results highlight the potential predictive value of admission lactate, prospective validation in more recent patient cohorts is warranted to confirm applicability in current settings.

## Conclusion

Higher initial lactate at ICU admission is associated with an increased risk of CS-POD and adverse postoperative outcomes. However, its predictive value is modest and should be interpreted cautiously as part of a broader clinical assessment rather than as an independent decision-making tool.

## Supplementary Information

Supplementary Material 1.

## Data Availability

We would like to clarify that the data utilized in this study were sourced from the eICU-CRD ([https://physionet.org/content/eicu-crd]). eICU-CRD are publicly available repositories containing de-identified electronic health records from critically ill patients.
