# Supplementary material for: Admission lactate at ICU entry and the risk of postoperative delirium after cardiac surgery: a retrospective cohort study using the eICU-CRD database
Source: BMC Cardiovasc Disord. 2026 May 7;26:548. doi: 10.1186/s12872-026-05915-5 (PMC13322019; doi:10.1186/s12872-026-05915-5)

Table S1. ICD-9 and ICD-10 codes for identifying cardiac surgeries and delirium.

	ICD-9 codes	ICD-10 codes
Cardiac surgeries	3511, 3512, 3514, 3521, 3522, 3523, 3524, 3527, 3611, 3612, 3613, 3614, 3615, 3616, 3619, 3845	0210089, 0210093, 0210099, 021009W, 02100A3, 02100A8, 02100A9, 02100AW, 02100Z3, 02100Z8, 02100Z9, 0211093, 0211099, 021109W, 02110A9, 02110AW, 02110Z3, 02110Z8, 02110Z9, 0212093, 0212099, 021209W, 02120AW, 02120Z8, 02120Z9, 0213093, 0213099, 021309W, 02130A3, 02130Z8, 02QF0ZZ, 02QG0ZZ, 02RF08Z, 02RF0JZ, 02RF0KZ, 02RG08Z, 02RG0JZ, 02RG0KZ, 02RJ08Z, 02RW0JZ, 02RX08Z, 02RX0JZ
Delirium	29281, 2930, 2931, 2939, 34831, 34982, 78009, 78097	F05, G92, G9341, R410, R4182

Table S2. Variance Inflation Factors (VIFs) for Assessment of Multicollinearity Among Covariates

Variable	VIF
lactate	1.19725779388422
age	1.30082168519785
BMI	1.25257212205768
gender	1.1576249069411
ethnicity	1.14508231258157
hypertension	1.10217045117723
myocardial_infarct	1.15791491848395
congestive_heart_failure	1.21899061956901
peripheral_vascular_disease	1.15903226275822
cerebrovascular_disease	1.31403758505124
chronic_pulmonary_disease	1.17782557499853
mild_liver_disease	1.1082237974384
diabetes_all	1.18348007778301
renal_disease	1.61815306103727
malignant_cancer	1.06923096023255
stroke	1.318792172
creatinine_max	2.15652083541048
chloride_max	1.42337936620936
hemoglobin_min	1.38494625757015
platelet_min	1.80054475940533
bun_max	1.67921312297081
sofatotal	2.64920331830674
apache_iv	2.18345152019047
hemodialysis	1.55327133435442
IABP	1.16924057786064
opioid	1.09151457191893

Note: VIF, Variance Inflation Factor, is used to assess the degree of multicollinearity among independent variables in regression models.

Table S3. Comparison of Baseline Characteristics Between Included and Excluded Patients

**Variables**	**Included Patients (n=358)**	**Excluded (n=5638)**	**P value**
Age (years)	65.8 [57.0, 74.0]	66.5 [58.0, 75.0]	0.182
BMI (kg/m²)	28.05 [24.40, 33.10]	27.60 [24.00, 32.50]	0.214
Male	245 (68.4%)	3798 (67.4%)	0.701
Ethnicity			0.633
White	223 (62.3%)	3470 (61.5%)	
Black	81 (22.6%)	1325 (23.5%)	
Other/Unknown	54 (15.1%)	843 (15.0%)	
Type of Surgery			0.091
CABG	190 (53.1%)	3125 (55.4%)	
Valve surgery	143 (39.9%)	2090 (37.1%)	
Combined surgery	25 (7.0%)	423 (7.5%)	
Congestive heart failure	78 (21.8%)	1085 (19.2%)	0.176
Diabetes	109 (30.4%)	1690 (30.0%)	0.872
Renal disease	64 (17.9%)	880 (15.6%)	0.189
Creatinine (mg/dL)	1.12 [0.87, 1.45]	1.08 [0.85, 1.39]	0.248
Hemoglobin (g/dL)	8.60 [7.30, 10.10]	8.90 [7.60, 10.40]	0.071
Platelets (×10⁹/L)	120.0 [88.0, 160.0]	125.0 [95.0, 170.0]	0.094
BUN (mg/dL)	18.5 [14.0, 25.5]	17.5 [13.0, 24.0]	0.133
APACHE IV	61.0 [44.0, 81.0]	59.0 [42.0, 78.0]	0.062
SOFA	5.5 [3.5, 7.5]	5.0 [3.0, 7.0]	0.058
Initial lactate (mmol/L)	2.10 [1.40, 3.30]	2.00 [1.30, 3.10]	0.089
In-hospital mortality	20 (5.6%)	265 (4.7%)	0.412

Table S4. Complete Case Sensitivity Analysis

Model	OR	Lower 95% CI	Upper 95% CI	P value
Model I	1.57	1.31	1.89	<0.001
Model II	1.52	1.26	1.85	<0.001
Model III	1.44	1.17	1.78	<0.001
Model IV (Full adjusted)	1.36	1.11	1.70	0.004

Figure. S1 Proportion of Missing Data Across Variables

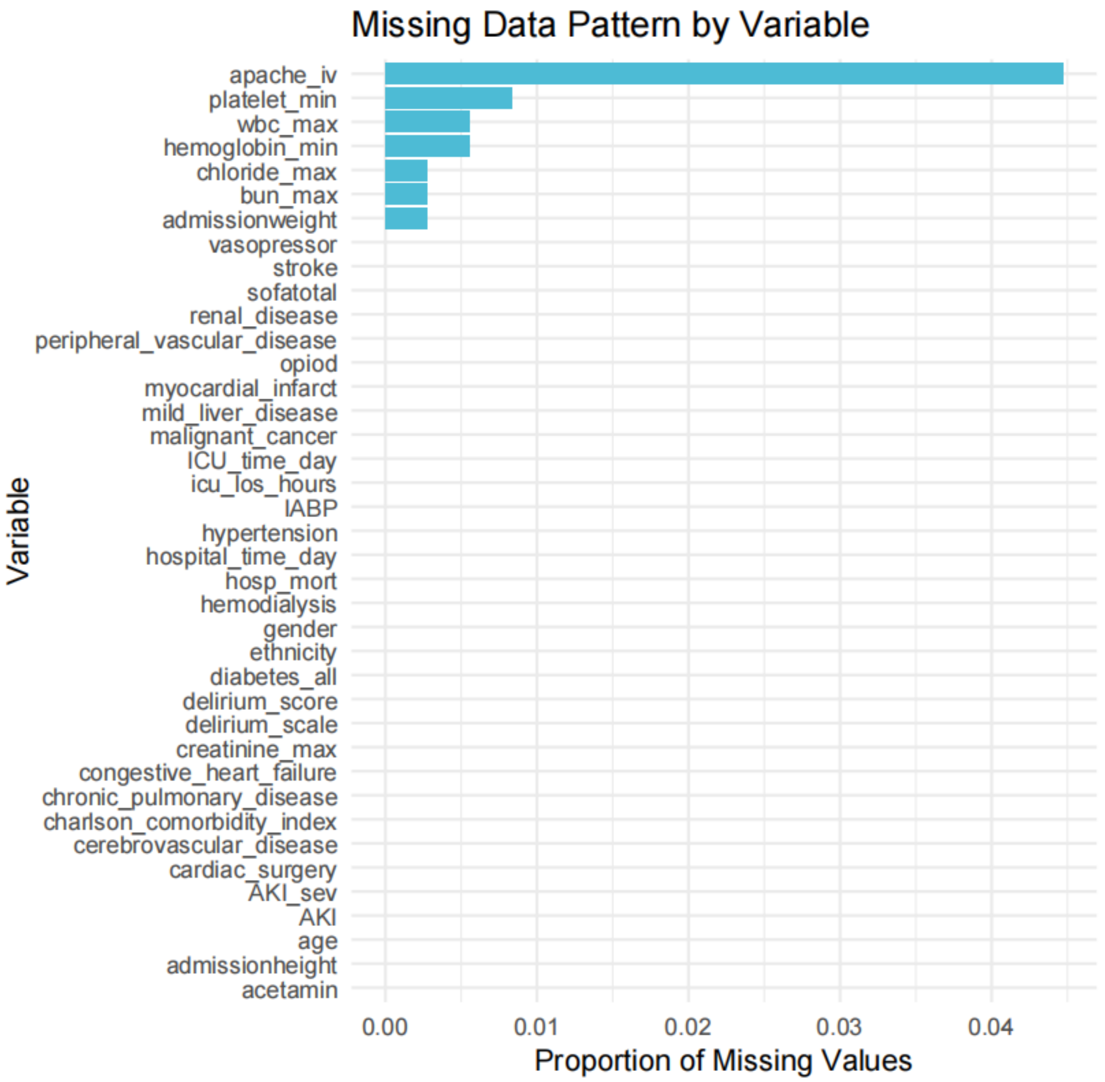

Supplement: Supplementary file 1 — Supplementary Material 1. [file 12872_2026_5915_MOESM1_ESM.pdf]
